# Optimized creation of glioblastoma patient derived xenografts for use in preclinical studies

**DOI:** 10.1186/s12967-017-1128-5

**Published:** 2017-02-09

**Authors:** Doreen William, Christina Susanne Mullins, Björn Schneider, Andrea Orthmann, Nora Lamp, Mathias Krohn, Annika Hoffmann, Carl-Friedrich Classen, Michael Linnebacher

**Affiliations:** 1 Children’s Hospital, University Medicine Rostock, Ernst-Heydemann-Str. 8, 18057 Rostock, Germany; 2 Department of Surgery, Molecular Oncology and Immunotherapy, University Medicine Rostock, Schillingallee 35, 18057 Rostock, Germany; 3 Institute of Pathology, University Medicine Rostock, Strempelstr. 14, 18057 Rostock, Germany; 4 Experimental Pharmacology and Oncology Berlin-Buch GmbH, Robert-Roessle-Str. 10, 13125 Berlin-Buch, Germany

**Keywords:** Glioblastoma multiforme, PDX, Preclinical mouse models, Therapy, Engraftment rate

## Abstract

**Background:**

Glioblastoma multiforme (GBM) is the most common and lethal brain tumor in adults, highlighting the need for novel treatment strategies. Patient derived xenografts (PDX) represent a valuable tool to accomplish this task.

**Methods:**

PDX were established by implanting GBM tissue subcutaneously. Engraftment success was compared between NMRI Foxn1^nu^ and NOD/SCID as well as between fresh and cryopreserved tissue. Established PDX were analyzed histologically and molecularly. Five PDX were experimentally treated with different drugs to assess their potential for preclinical drug testing.

**Results:**

Establishment of PDX was attempted for 36 consecutive GBM cases with an overall success rate of 22.2% in NMRI Foxn1^nu^ mice. No difference was observed between fresh or cryopreserved (20–1057 days) tissue in direct comparison (n = 10 cases). Additionally, engraftment was better in NOD/SCID mice (38.8%) directly compared to NMRI Foxn1^nu^ mice (27.7%) (n = 18 cases). Molecular data and histology of the PDX compare well to the primary GBM. The experimental treatment revealed individual differences in the sensitivity towards several clinically relevant drugs.

**Conclusions:**

The use of vitally frozen GBM tissue allows a more convenient workflow without efficiency loss. NOD/SCID mice appear to be better suited for initial engraftment of tumor tissue compared to NMRI Foxn1^nu^ mice.

**Electronic supplementary material:**

The online version of this article (doi:10.1186/s12967-017-1128-5) contains supplementary material, which is available to authorized users.

## Background

Glioblastoma multiforme (GBM) is the most common primary brain tumor in adults [1, 2]. With a median survival of 14–16 months from diagnosis, the prognosis for GBM patients is very dismal and novel therapeutic strategies are urgently needed to combat this disease [3–6]. A promising approach is to further facilitate personalized therapy regimens, which are tailored to specific molecular alterations of individual tumors. Besides methylation status of the MGMT promoter, established prognostic parameters for GBM are missing [7]. However, with next generation sequencing techniques on the rise, more detailed analyses of cancer genomes are becoming routinely available to clinicians and researchers worldwide [8, 9]. For example, previous analyses of GBM genomes revealed several common alterations in tyrosine kinase signaling (e.g. mutations or amplification of EGFR, ERBB2, PDGFRA, MET and PTEN) [10]. Several tyrosine kinase inhibitors and other targeted drugs entered clinical trials for GBM treatment (e.g. vandetanib, bevacizumab, nimotuzumab) however, with so far unsatisfying outcome [11–13].

In order to accomplish better personalized therapy strategies, individual models of GBM and sufficient amounts of tumor material for detailed molecular and functional analyses are required.

Although the generation of individual in vitro models of GBM is feasible with success rates of cell culture establishment of approximately 60% [14], in vitro models have several disadvantages. Due to enhanced clonal selection in vitro, the use of (ultra-) low passage cell lines is mandatory to best preserve intratumoral heterogeneity [15]. Additionally, during in vitro culture, several genomic aberrations, e.g. amplification of *EGFR*, which are present in the primary tumor, are not maintained [16, 17]. In contrast, intratumoral heterogeneity and genomic aberrations are well maintained in heterotopic or orthotopic patient derived in vivo models of GBM [18, 19]. The establishment of such PDX models requires optimized logistics and standardized protocols; further the combined expertise from different fields (surgery, molecular biology and animal care facility) is imperative. In this study, we present a feasible method for the establishment of GBM PDX models from patient tumor material. First, we investigated the success rates of PDX establishment using cryopreserved GBM tissue (postoperative immediately frozen) compared to fresh tumor tissue. We could demonstrate that cryopreservation with subsequent long term storage of GBM tissue at ultra-low temperatures is a suitable and logistically convenient method for xenografting at a later time point. Furthermore, we compared the success rates of PDX establishment using two different immunocompromised mouse strains (NMRI Foxn1^nu^ and NOD/SCID) in order to further optimize PDX creation. Once established, PDX models may be a suitable tool for the prediction of therapy outcomes as well as planning of patient individual treatments in order to identify the best possible therapy option for patients suffering from GBM.

## Methods

### Tumor specimen collection and cryopreservation

Tumor tissue was collected directly from the operation theater at the department of neurosurgery at the University Medicine of Rostock. Specimen collection was conducted in accordance with the ethics guidelines for the use of human material, approved by the Ethics Committee of the University of Rostock (Reference number: A 2009/34) and with informed written consent from all patients prior to surgery. Tumor tissue was cut with sterile scalpels to small tissue cubes (approximately 3 × 3 × 3 mm). For cryopreservation, 4 tumor cubes were transferred to a sterile cryo-tube containing 1.5 mL freezing medium (fetal calf serum, 10% DMSO) and immediately frozen at −80 °C in a freezing container. Vitally frozen tumor material was transferred after overnight cooling at −80 °C into liquid nitrogen for long term storage at ultra-low temperatures.

### Xenografting

Tumor tissue cubes were implanted subcutaneously into the flanks of female 6–8 weeks old NMRI Foxn1^nu^ or NOD/SCID mice under anaesthesia (Ketamine/Xylazin 90/6 mg/kg Bw) as previously described [20–22]. Briefly, cryopreserved tumor tissue was thawed at 37 °C and washed with PBS prior to subcutaneous implantation.

Care and housing of the animals was provided at the animal facilities of the University Medicine Rostock in accordance with recommendations from the Guide for the Care and Use of Laboratory Animals of the National Institutes of Health. The procedure was approved by the Committees on the Ethics of Animal Experiments (Landesamt für Landwirtschaft, Lebensmittelsicherheit und Fischerei Mecklenburg-Vorpommern; permission number: 7221.3-1.1-083/11). Mice were kept in a specific pathogen free environment and exposed to 12 h light/12 h darkness cycles with standard food and water (supplemented with Co-trimoxazol for 6 weeks after surgery) ad libitum. Mice were sacrificed when tumors grew to a volume of 1 cm^3^ and tumor material was collected for further studies or passaged further in NMRI Foxn1^nu^ mice.

For the establishment of orthotopic GBM PDX, a single cell suspension derived from previously established GBM PDX was injected into the brain of NOD/SCID mice. Upon first signs of extracerebral tumors at place of cell injection or abnormal behavior, the mice were sacrificed and the brains were snap frozen for further analysis.

### Experimental treatment of tumor bearing mice

The chemotherapeutic response of the PDX models was determined in female NMRI nu/nu mice (Janvier, Le Genest-Saint-Isle, France). Once tumors became palpable, tumor size and body weight were measured twice a week. Tumor volumes (V) were calculated by the formula V = (length × width^2^)/2 and related to the values at the first day of treatment (relative tumor volume, RTV). Median treated to control (T/C) values of RTV was used for the evaluation of each treatment modality.

When the mean tumor volume reached the indicated starting volume (80–120 mm^3^), mice were randomized to the six treatment arms (five mice per group) and treatment was started. If not mentioned otherwise, the following drugs and modalities were used in the single treatment studies: everolimus 5 mg/kg, orally, (days 1–5) × 2; sorafenib 80 mg/kg, orally, (days 1–5) × 2; bevacizumab 10 mg/kg, intraperitoneally, (three times a week) × 2; irinotecan 15 mg/kg, intraperitoneally, days 1–5; salinomycin 10 mg/kg, orally, days 1–14; temozolomide 90 mg/kg, orally, days 1–5. Control mice were treated with the vehicle alone (saline), orally. Doses and schedules were chosen according to previous experience in animal experiments and represent the maximum tolerated or efficient doses. The injection volume was 0.2 mL/20 g body weight.

### EGFR copy number analysis

Genomic DNA (gDNA) from snap frozen tumor tissue was isolated using the Wizard Genomic DNA Purification Kit (Promega, Mannheim, Germany) according to the manufacturer’s instructions. EGFR copy number was determined by quantitative PCR on a StepOne Realtime PCR system (Applied Biosystems, Darmstadt, Germany) with SensiFastSYBR Hi-Rox-Kit (Bioline, Luckenwalde, Germany) in triplicates. Commercial normal human gDNA (Promega) served as calibrator and the repetitive element LINE1 as endogenous control. The EGFR copy number was calculated with the $^{\Delta \Delta } {\text{C}}_{\text{t}}$-algorithm.

### MGMT promoter methylation analysis

MGMT promoter methylation was analyzed with the MethyLight method [23]. Briefly, gDNA was subject to bisulfide conversion using the Epitect Bisulfite Kit (Qiagen, Hilden, Germany) according to the manufacturer’s recommendations. Quantitative PCR was performed with the SensiFast Probe HiRox Kit (Bioline) and a primer/probe combination specific for the methylated MGMT promoter sequence (Additional file 1). Fully methylated SSSI treated DNA served as calibrator and the collagenase gene 2A1 (COL2A1) served as endogenous control. The percentage of methylated reference (PMR) value was calculated by dividing the MGMT/COL2A1 ratio of the sample by the MGMT/COL2A1 ratio of the SSSI-treated DNA multiplied by 100. Samples with a PMR value >4 were considered as methylated.

### Mutation analysis

All samples were analyzed for mutations in the following loci: IDH1 R132 (exon 4), IDH2 R172 (exon 4), BRAF V600 (exon 15), KRAS G12, G13 (exon 2) and Q61 (exon 3) and TP53 exons 5–8. The desired genomic regions were amplified by PCR (Additional file 1). The PCR products were purified and used as template for Sanger sequencing using BigDye^®^ Terminator v1.1 Cycle Sequencing Kit (Applied Biosystems) according to the manufacturer’s protocol. The sequencing products were purified using the BigDye XTerminator^®^ Purification Kit and analysed with the 3500 genetic analyzer system using the SeqScape^®^ Software v2.7 (all Applied Biosystems).

### Genetic fingerprint analysis

A genetic fingerprint analysis was performed by PCR using 9 different loci (D5S818, D7S820, D16S539, D13S317, Amelogenin, vWA, TPOX, TH01 and CSF1; Additional file 1) to verify the identity of the PDX in comparison to original GBM material [24]. Briefly, 25 ng DNA of each sample were used in 2 multiplex PCR reactions (cycling conditions: initial denaturation at 96 °C for 2 min, 30 cycles of 94 °C 30 s, 59 °C 2 min and 72 °C 1.5 min, 60 °C for 45 min). PCR products were diluted tenfold and subsequently analyzed by capillary electrophoresis.

### Immunohistochemistry and H&E-staining

Formaldehyde fixed tissue samples were processed using the ExcelsiorAS system (ThermoScientific) according to the manufacturer’s recommendations. For GFAP immunohistochemistry a ready to use anti-GFAP primary antibody (Dako, Hamburg, Germany) was used and the samples were processed on an automated system, EnVision™ FLEX (Dako), following manufacturer’s instructions. Hematoxylin and Eosin staining was performed following established standard protocols [25].

### Statistics

Statistical analysis was performed using SigmaPlot 10.0 (Systat Software). Tumor volumes were compared between treatment and control groups at the end of the experiment and analyzed by unpaired *t* test.

## Results

### Engraftment of primary tumor samples in immunocompromized mice

Overall, 42 tumor samples were collected and subsequently cryopreserved. Out of all samples 36 samples were classified as GBM (WHO°IV), 5 as astrocytomas (WHO°I–III) and 1 an anaplastic oligodendroglioma (WHO°III). Information on patient characteristics, diagnosis, and molecular alterations of the tumors is summarized in Table 1 and Additional file 2.

**Table 1:** Overview of GBM patient characteristics, molecular alterations, cryoperiod of the samples prior to subcutaneous implantation and outcome of PDX establishment attempts in NMRI Foxn1^nu^ mice

Sample ID	Sex/age	Molecular alterations	Cryoperiod (days)	Engraftment
HROG02	M/68	P53 (R248Q), 3xEGFR, MGMT(M)	305	–
HROG04	F/53	36xEGFR, MGMT(U)	270	–
			588	–
			826	–
HROG05	F/60	82xEGFR, K-ras (G12D), MGMT(M)	268	✓
HROG06	M/53	P53 (R273H, R306*), 82xEGFR, MGMT(U)	260	–
			570	✓
HROG07	M/55	12xEGFR, MGMT(U)	143	–
			699	–
HROG10	M/74	MGMT(U)	71	–
			627	–
HROG11	F/54	P53 (R248Q), 3xEGFR, MGMT(U)	56	–
HROG12	M/64	36xEGFR, MGMT(U)	307	✓
HROG13	F/77	MGMT(U)	318	–
			529	✓
HROG15	M/56	n.d.	240	–
HROG16	M/53	MGMT(U)	237	–
HROG17	M/70	3xEGFR, MGMT(M)	194	✓
HROG19	M/69	8xEGFR, MGMT(U)	217	–
HROG21	M/44	IDH1 (R132H), MGMT(U)	192	–
HROG22	M/66	MGMT(M)	167	–
HROG23	F/60	BRAF (V600E), MGMT(U)	191	–
			1057	–
HROG24	F/73	P53 (R273C), 42xEGFR, MGMT(U)	112	–
			350	–
HROG25	F/77	MGMT(U)	117	–
HROG31	F/59	MGMT(U)	55	–
			214	–
HROG32	F/76	44xEGFR, MGMT(U)	125	–
HROG33	F/46	31xEGFR, MGMT(U)	119	✓
HROG34	F/69	96xEGFR, MGMT(U)	133	–
HROG36	F/80	MGMT(U)	80	–
HROG38	F/49	MGMT(U)	58	–
			236	–
HROG41	M/71	IDH1 (R132H), MGMT(M)	31	–
			209	–
HROG42	F/70	MGMT(U)	30	–
			189	–
HROG49	M/45	MGMT(U)	Fresh	–
			360	–
HROG52	M/47	n.d.	Fresh	✓
			308	–
HROG54	M/58	MGMT(M)	Fresh	–
			281	–
HROG55	F/74	MGMT(M)	Fresh	–
			278	–
HROG56	F/76	MGMT(U)	Fresh	–
			222	–
HROG58	F/57	MGMT(U)	Fresh	–
			165	–
HROG59	M/60	16xEGFR, MGMT(U)	Fresh	✓
			152	–
HROG60	M/51	2xEGFR, MGMT(U)	Fresh	–
			126	–
			513	–
HROG63	M/48	18xEGFR, MGMT(U)	Fresh	–
			20	–
HROG64	F/57	MGMT(M)	Fresh	–
			20	–

*M* male, *F* female, *xEGFR* EGFR gene amplification, *MGMT(M)* methylated MGMT promoter, *MGMT(U)* unmethylated MGMT promoter, *n.d.* not determined

Cryopreserved tissue samples of all cases were implanted bilaterally subcutaneously in the flanks of female 6–8 weeks old NMRI Foxn1^nu^ mice. Cryopreservation periods of the tumor samples ranged from 20 to 1057 days. Engraftment of frozen GBM samples was successful in 8 out of 36 cases (22.2%) (Table 1). Engraftment of the 5 astrocytoma samples and the anaplastic oligodendroglioma sample was not successful (Additional file 2).

For 10 GBM cases, a direct comparison of tumor take rate between cryopreserved tumor tissue and tumor tissue freshly received from the operation theater was performed (Table 1). Engraftment of cryopreserved tumor tissue in NMRI Foxn1^nu^ mice was successful in one case out of 10 (HROG52, 10.0%); identical to engraftment of fresh tumor tissue (HROG59, 10.0%). In 2 cases the initial tumor growth of fresh GBM tissue was followed by complete spontaneous regression (HROG58 and HROG60). Thus, within this limited number of cases, there was no difference between engraftment success of cryopreserved and fresh GBM tissue samples. Furthermore, success rates of engraftment in NMRI Foxn1^nu^ mice or in NOD/SCID mice were compared on the basis of 18 cryopreserved GBM samples (Table 2). In NMRI Foxn1^nu^ mice, 5 out of 18 samples were successfully engrafted (27.7%). The success rate was higher in NOD/SCID mice (7 out of 18 samples; 38.8%). Four cases (HROG05, HROG06, HROG13 and HROG17) were successfully engrafted in both mouse strains, two cases could only be successfully engrafted in NOD/SCID mice and one case only engrafted in NMRI Foxn1^nu^ mice (Table 2).

**Table 2:** Direct comparison of PDX establishment success between NMRI Foxn1^nu^ and NOD/SCID mice

Sample ID	Cryoperiod (days)	NMRI Foxn1^nu^	NOD/SCID
HROG02	305–1963	–	–
HROG04	270–1213	–	–
HROG05	268–1248	✓	✓
HROG06	260–1918	✓	✓
HROG07	143–1123	–	✓
HROG10	71–1763	–	–
HROG13	318–1634	✓	✓
HROG15	240–1570	–	–
HROG17	194–1555	✓	✓
HROG19	217–1570	–	–
HROG21	192–900	–	–
HROG22	167–875	–	✓
HROG23	191–1057	–	–
HROG24	112–1494	–	–
HROG25	177–912	–	–
HROG38	58–1500	–	✓
HROG49	0–638	–	–
HROG59	0–427	✓	–

Overview of PDX establishment success of 18 cases, checkmarks indicate successful engraftment

### Long-term stability of GBM PDX models

All initially successfully engrafted cases, both in NMRI Foxn1^nu^ and NOD/SCID mice, were passaged in vivo to determine if these PDX models demonstrate stable growth behavior. PDX models initially established in NOD/SCID mice (Table 2) were transferred in NMRI Foxn1^nu^ mice (Additional file 3). 9 out of the 11 initially positive PDX cases (81.8%) were successfully engrafted in the first in vivo transfer and subsequently passaged further. Two initially positive cases (HROG12 and HROG52) were unable to form a tumor in subsequent in vivo transfers into NMRI Foxn1^nu^ mice. In the case of HROG12, a second in vivo transfer attempt also failed. To date, 6 GBM PDX cases reached a minimum of 5 in vivo passages and therefore are considered as long-term stable PDX models (Additional file 3). The remaining cases HROG17, HROG33 and HROG38 have reached 3 or 4 passages and thus will very likely become long-term stable PDX models as well. Overall, a strong trend towards accelerated tumor growth was observed with increasing in vivo passage number (Table 3).

**Table 3:** Analysis of MGMT promoter methylation and EGFR amplification of GBM tumors and corresponding PDX over several in vivo passages

Sample ID	MGMT promoter methylation (PMR)	EGFR amplification	Tumor volume >0.2 cm^3^ at day
*HROG05*			
Tumor	M (34,5)	82×	–
PDX P2	U (0)	12×	60
*HROG06*			
Tumor	U (0)	82×	–
PDX P0	U (0)	75×	54
PDX P1	U (0)	69×	24
PDX P2	U (0)	103×	40
PDX P3	U (0)	123×	27
PDX P4	U (0)	144×	25
PDX P5	U (0)	147×	19
*HROG07*			
Tumor	U (0)	12×	–
PDX P2	U (0)	152×	82
PDX P4	U (0)	96×	98
*HROG12*			
Tumor	U (1,4)	37×	–
PDX P0	U (0)	52×	123
*HROG13*			
Tumor	U (3,9)	1×	–
PDX P1	M (4)	2×	25
PDX P2	M (5)	2×	31
PDX P3	M (15)	2×	46
*HROG17*			
Tumor	M (14)	4×	–
PDX P2	M (11)	1×	59
*HROG22*			
Tumor	M (22,2)	1×	–
PDX P0	M (6)	2×	158
PDX P1	M (22)	2×	54
PDX P3	M (73)	1×	46
PDX P5	M (97)	2×	26
*HROG33*			
Tumor	U (0)	31×	–
PDX P1	U (0)	67×	90
*HROG59*			
Tumor	U (0)	16×	–
PDX P2	U (0)	85×	68
PDX P3	U (0)	92×	31
PDX P4	U (0)	47×	31
PDX P5	U (0)	36×	54

*M* methylated, *U* unmethylated, *PMR* percentage of methylated reference

### Morphology of primary GBM and their PDX derivates is conserved over several in vivo passages

**Fig. 1:**
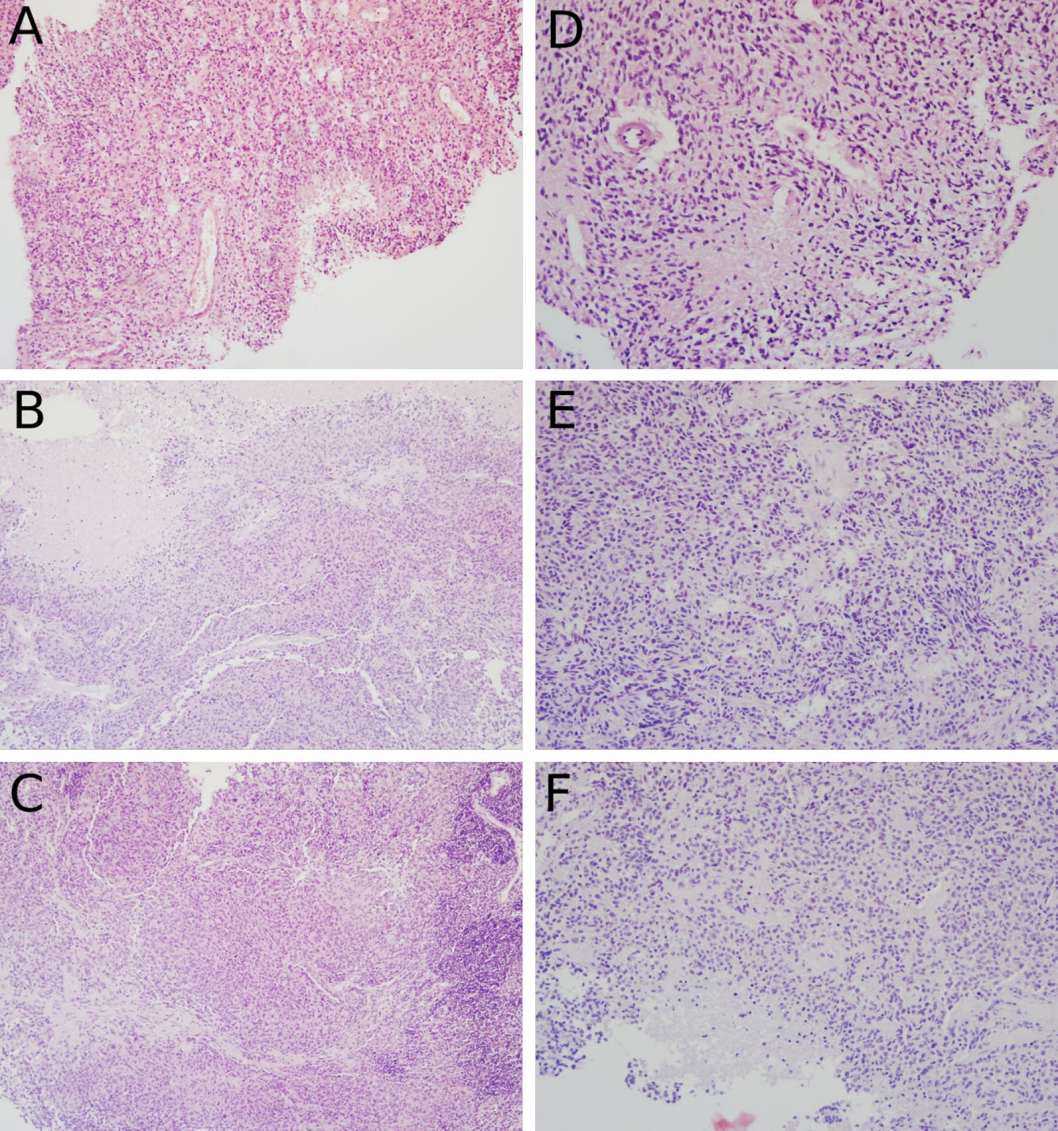
Hematoxylin and eosin staining of paraffin embedded GBM and PDX sections. **A** HROG33 primary GBM, **B** HROG33 PDX after first in vivo transfer, **C** HROG33 PDX after second in vivo transfer, **D** HROG59 primary GBM, **E** HROG59 PDX after first in vivo transfer, **F** HROG59 PDX after second in vivo transfer. 200-fold magnification

As shown for HROG33 and HROG59 in two consecutive in vivo passages (designated as “PDX-T1” and “PDX-T2”), the PDX models resemble the respective primary GBM closely (Fig. 1). The HROG33 PDX models show a slightly higher compactness of cell structure than the primary tumor, but important characteristics such as content of mitotic cells, degree of pleomorphy and necrosis compare well with the primary tumor. Albeit the limited sample size of the PDX, both passages of HROG33 PDX fulfilled formal requirements which would have allowed a correct GBM diagnosis. The HROG59 PDX models also show key characteristics of the primary tumor (high degree of pleomorphy, necrosis and content of hyperchromatic cells). Of the two HROG59 PDX transfers, only HROG59 PDX-T2 formally fulfils the requirements for a GBM diagnosis. The specific section of HROG59 PDX-T1 does–most likely due to the small sample size—not contain a thrombotic blood vessel and thus, does not fulfil all formally required characteristics for a GBM diagnosis; yet compares very well to the primary tumor otherwise.

In general, the PDX models show a highly similar morphology between first and second in vivo passage. As compared to the respective primary GBM, important characteristics were conserved in the PDX models in these low passages. However, the heterotopic PDX do not show invasive growth behavior, which is characteristic for GBM. Invasive growth into surrounding tissue was observed in orthotopic models of two exemplary cases (HROG06 and HROG59; Additional file 4). High expression of glial fibrillary acidic protein (GFAP), a marker for astrocytic cells, is evident in both primary tumors and the corresponding heterotopic PDX models in both passages (Additional file 5). This additionally verifies that the GBM PDX models conserve their neuronal character.

### Comparison of molecular aberrations between primary GBM and corresponding PDX

The presence of mutations found in the primary tumor was analyzed in every corresponding PDX model (Table 1; Additional file 2). In all cases, the mutations in the genes K-RAS, P53 and IDH1 could be confirmed in the PDX models. However, analyses of MGMT promoter methylation status of primary GBM and derived PDX models revealed differences in 2 out of 9 cases (Table 3). Furthermore, genomic amplification of EGFR was variable in the PDX models over several in vivo passages as well as in comparison to the primary GBM in all cases with EGFR amplification in the primary tumor. GBM cases without EGFR amplification did not gain additional EGFR gene copies over the PDX passages. Identity of all PDX models was verified by genetic fingerprint analyses (Additional file 6) and matched in all cases.

### Experimental therapy of GBM PDX models

**Fig. 2:**
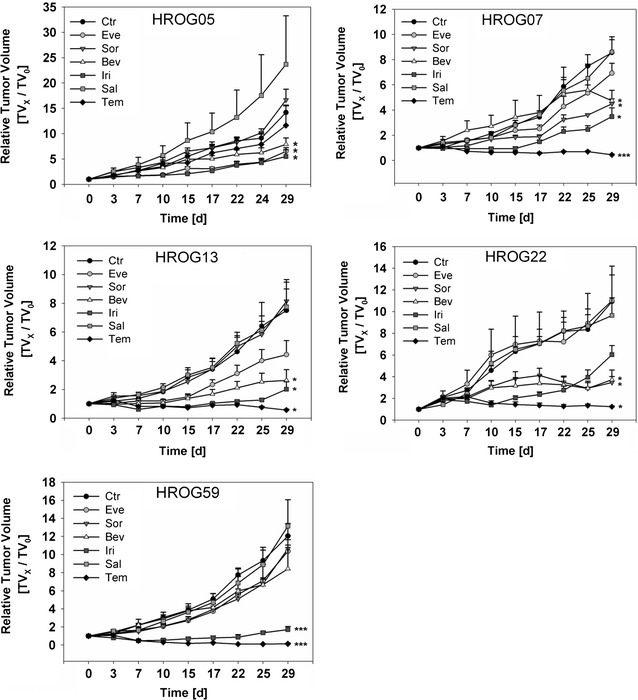
Experimental treatment outcomes of 5 GBM PDX. Development of relative tumor volumes over time, error bars indicate the standard errors of the mean. *p < 0.005, ***p < 0.001, p-values were calculated with a t test at the end of the experiment in comparison to untreated controls. *ctr* control treated with PBS, *Eve* everolimus, *Sor* sorafenib, *Bev* bevacicumab, *Iri* irinotecan, *Sal* salinomycin, *Tem* temozolomide

Five different GBM PDX models were experimentally treated with monotherapies of temozolomide, everolimus, sorafenib, salinomycin, bevacizumab or irinotecan (5 mice per group). Control mice were treated with physiological saline solution. 4 out of 5 PDX models were highly susceptible to temozolomide monotherapy, only HROG05 showed intrinsic temozolomide resistance (Fig. 2). Good treatment results were also obtained with the anti-VEGF antibody bevacizumab, which had a positive effect in all cases tested. Irinotecan, a topoisomerase inhibitor, was effective in 3 cases (HROG05, HROG13 and HROG59). Treatment with the mTOR inhibitor everolimus had positive effects in 2 cases (HROG05 and HROG13). The multi-kinase inhibitor sorafenib was effective only in one case (HROG22) and salinomycin treatment had no effect in all cases tested. Additionally, all experimentally treated PDX models were analyzed for potentially relevant mutations by panel sequencing covering 212 target regions in 48 cancer related genes (Table 4).

**Table 4:** Mutations in PDX models used for in vivo therapy experiments

Sample ID	Mutations in PDX
HROG05	EGFR (R108K, Y626H), K-Ras (G12D), P53 (R280K)
HROG07	APC (A1340 V), FLT3 (V592I), PIK3CA (E545K), PTPN11 (S502L)
HROG13	ABL1 (A288S), ATM (F858L), ERBB2 (G748C), GNA11 (N336K), PTEN (S207C), VHL (E94*)
HROG22	PIK3CA (E545K), PTPN11 (S502L)
HROG59	ERBB2 (Del fs*), GNAQ (2x Del fs*), KDR (Q472H), PTEN (Q17L, M198I, L265I)

Mutations identified by amplicon panel sequencing covering 212 target regions in 48 cancer-related genes (Illumina MiSeq TSACP (Illumina Variant Caller 3.1.10.0)). Del: deletion, fs*: frame shift leading to stop, *: stop gain

## Discussion

Glioblastoma multiforme remains a tumor difficult to treat with a very dismal prognosis. Hence, gaining a better understanding of molecular characteristics of individual GBM is mandatory for the development of individualized therapy strategies. This task requires sufficient amounts of tumor material for analysis and—potentially—therapy response prediction approaches. Individual GBM PDX recommend themselves for this purpose, since tumor material can be propagated for further studies in an in vivo environment, while maintaining intratumoral heterogeneity as well as most genomic aberrations [26]. However, establishment of GBM PDX requires optimized logistics and standardized protocols. We demonstrate here that GBM tissue cryopreserved and subsequently stored for longer time periods enables xenografting at a later time point. Additionally, and to the best of our knowledge, this is the first study in which xenografting of fresh and vitally frozen GBM tissue was compared directly. We did not observe a difference of engraftment success between fresh and vitally frozen GBM tumor material. Hence, many logistic obstacles (e.g. transport conditions and transport time of tumor tissue, availability of mice for xenografting, opening hours at laboratory animal facilities) can be circumvented by using this simple and feasible method of GBM tissue cryopreservation. However, we did not observe successful engraftment of both fresh and vitally frozen GBM tissue derived from an identical primary GBM tumor. Although this was not subject of further investigation, it seems likely that the quality of implanted tumor tissue—fresh or vitally frozen—as well as individual and yet unknown mouse factors have an influence on the success of an individual tumor graft [27].

Overall engraftment success in NMRI Foxn1^nu^ mice is with 22.2% rather low in comparison to successful establishment of GBM cell lines [14], orthotopic GBM PDX [18] or of PDX from other tumor entities [22], but well in line with previous reports from other studies on heterotopic GBM [28]. The GBM PDX establishment success rate appears to be higher in NOD/SCID mice, which lack B- and T-Lymphocytes as well as NK cells, than in NMRI Foxn1^nu^ mice (38.8 vs 27.7% in the directly compared 18 cases). Out of all 7 successfully engrafted cases in this cohort of 18 cases, 4 cases were positive in both mouse strains, 2 cases only engrafted in NOD/SCID mice and one case was only successful in NMRI Foxn1^nu^ mice. NMRI Foxn1^nu^ mice are a widely used host for tumor xenograft studies due to the complete absence of T-Lymphocytes, yet other components of the immune system (NK cells, granulocytes, monocytes/macrophages, dendritic cells and B-Lymphocytes) are still present. Although residual active immune cells in NMRI Foxn1^nu^ mice likely play a role in preventing tumor formation, other crucial parameters like the quality of the implanted tumor tissue, the biology of the individual tumors and the individual mice are likely to play an even greater role [27]. In three cases (HROG23, HROG58, HROG60), we observed initial tumor formation followed by complete regression of engrafted primary tumors in NMRI Foxn1^nu^ mice (data not shown) before material could be saved for subsequent in vivo passaging. In these cases, engrafting repetitions might still result in successful PDX generation as was the case for HROG06 and HROG13.

Mutations present in the primary GBM were maintained in all analyzed genes in all PDX models over several passages. However, genomic amplification of EGFR varied between primary GBM and their PDX in nearly all cases where EGFR amplification has been observed in the primary GBM. Although not further investigated, it seems likely that this effect is due to clonal selection processes as cells with high EGFR copy numbers are not evenly distributed throughout the whole tumor tissue [29]. However, we also observed variations in the EGFR copy numbers over the PDX passages, which implies that cell intrinsic factors are still active.

Nevertheless, PDX models of GBM are valuable tools for further studies, for development of novel therapeutics as well as response prediction attempts for individualized therapy approaches. Intratumoral heterogeneity is generally well maintained in these models and we could show that the morphology of a primary GBM tumor is comparable to its PDX over several passages. However, heterotopic PDX do not show invasive growth into surrounding tissue compared to orthotopic GBM models. Nevertheless, heterotopic (or orthotopic) GBM PDX models using cryopreserved tissue specimens are a suitable tool for therapy response prediction since this method is feasible to create sufficient amounts of tumor material by in vivo passaging in mice to allow systematic and standardized testing of several therapeutic substances in vivo. Responses towards clinically used therapeutics in our test series of GBM PDX models were as diverse as expected from previous studies but also from clinical experience [30, 31]. Generally good in vivo responses were obtained with temozolomide, irinotecan and bevacizumab. A combination of these drugs just recently proofed beneficial in some cases of unresectable GBM in the neoadjuvant setting in a clinical phase II trial [32]. Beside this, two out of five PDX models responded to sorafenib and one to everolimus. In total, these chemo-response data proof applicability and can be considered for targeted selection of these novel GBM PDX models in future preclinical studies either in the heterotopic or orthotopic setting.

## Conclusions

Despite aggressive treatment regimen, GBM remains a lethal brain tumor and development of new therapy strategies is an urgent task to combat this disease. In order to achieve targeted therapy regimen, establishment an analysis of individual in vivo models of GBM is essential. Although orthotopic PDX have the advantage of an appropriate tumor microenvironment, heterotopic models, as presented in this study, are of high value for several reasons. Heterotopic PDX enable the production of sufficient amounts of tumor tissue for extensive molecular and functional analyses. Furthermore, heterotopic PDX histologically and molecularly resemble the primary GBM closely and the establishment of those PDX is technically easily feasible. We demonstrated that long term cryopreserved GBM tissue can be engrafted in mice without loss of engraftment efficiency, which allows for a convenient workflow and improved logistics. We also systematically compared the PDX establishment success between two widely used mouse models, NMRI Foxn1^nu^ and NOD/SCID, for 18 GBM cases and found engraftment success to be higher in NOD/SCID mice (38.8 vs 27.7% in NMRI Foxn1^nu^ mice). However, factors such as quality of tumor tissue pieces and individual mice also play a role in overall engraftment success, but are not easily influenced by the user. Taken together, our data provide the means for optimized establishment of GBM PDX with regard to choice of mouse strain and use of cryopreserved tissue.

## Additional files

**Additional file 1.** Sequences of oligonucleotides and probes.Nucleotide sequences and labeling of all oligonucleotides and probes used in this study.

**Additional file 2.** Overview of patient characteristics diagnosed with WHO°I–III tumors. Summary of molecular alterations, cryoperiod of the samples prior to implantation and the outcome of PDX establishment attempts.

**Additional file 3.** Overview of highest in vivo passages of engrafted samples. Given numbers indicate the highest in vivo passage reached for given samples, 0 indicates failure at regrafting after initially positive engraftment from primary GBM tissue.

**Additional file 4.** Cresyl violet staining of representative orthotopic GBM PDX samples. GBM cells were injected intracranially to establish orthotopic GBM PDX models. A-D) HROG06 31d post injection of 2 × 10^5^ GBM cells, A & C) whole brain section for assessment of tumor volume and localization, B & D) 100× magnification for assessment of invasive growth of GBM cells into surrounding tissue; E & F) HROG59 34d post injection of 8.7 × 10^5^ GBM cells, F) 100x magnification; G & H) HROG59 34d post injection of 3.5 × 10^5^ GBM cells, H) 100x magnification.

**Additional file 5.** GFAP Immunohistochemistry staining of paraffin embedded GBM and PDX tissue sections. A) HROG33 primary GBM, stained with new fuchsine B) HROG33 PDX after first in vivo transfer, C) HROG33 PDX after second in vivo transfer, D) HROG59 primary GBM, E) HROG59 PDX after first in vivo transfer, F) HROG59 PDX after second in vivo transfer. 200-fold magnification B-F were stained with 3,3′-Diaminobenzidine.

**Additional file 6.** Results of genetic fingerprint analysis. Identity verification of all PDX cases over several in vivo passages by genetic fingerprint analysis.

## Abbreviations

GBM: glioblastoma multiforme
PDX: patient derived xenograft
RTV: relative tumour value
Bw: bodyweight
