# Supplementary figures and images for: Optimized creation of glioblastoma patient derived xenografts for use in preclinical studies
Source: J Transl Med. 2017 Feb 9;15:27. doi: 10.1186/s12967-017-1128-5 (PMC5301415; doi:10.1186/s12967-017-1128-5)

## Slide 1
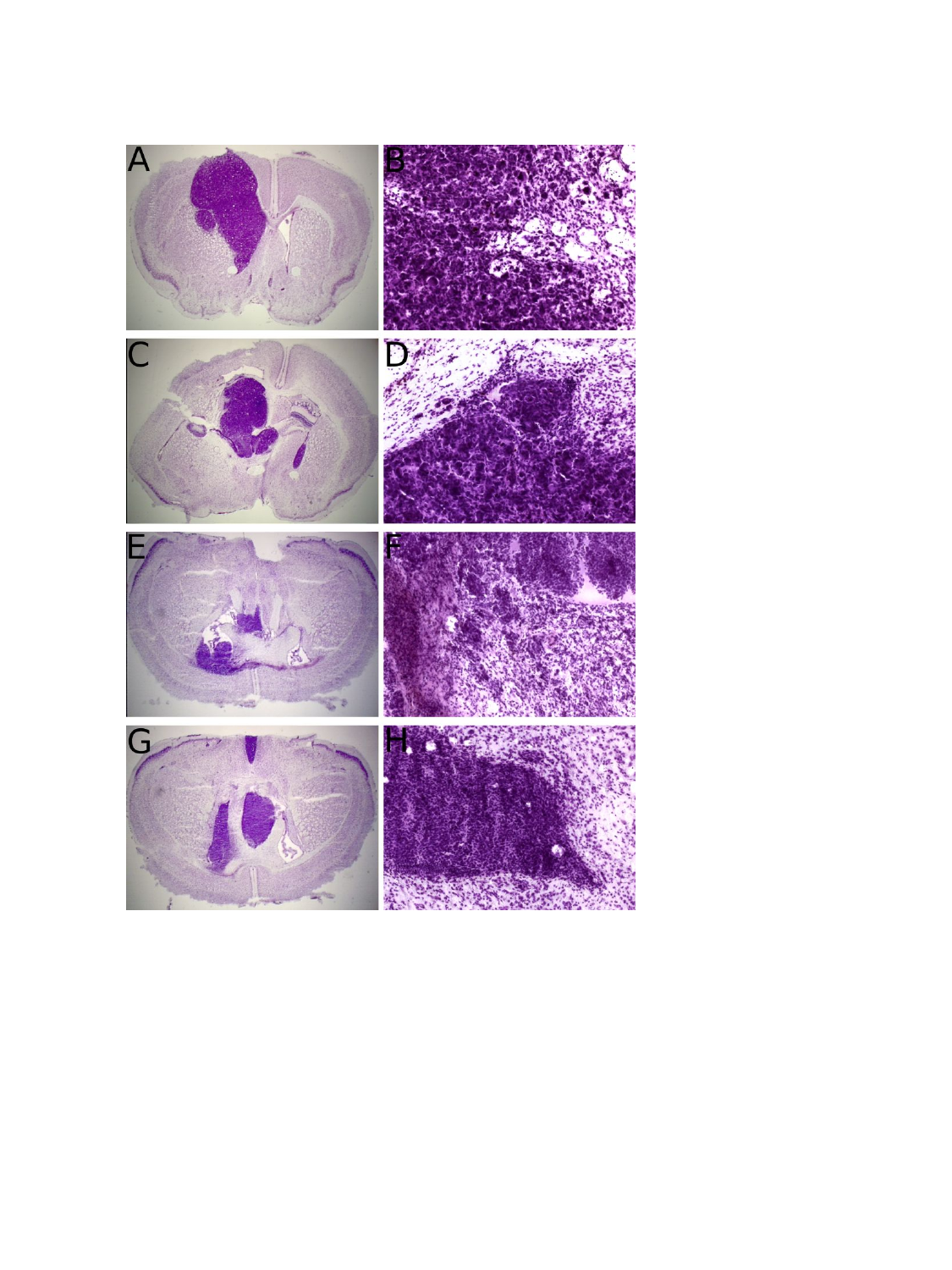

Supplement: Supplementary file 4 — Additional file 4. Cresyl violet staining of representative orthotopic GBM PDX samples. GBM cells were injected intracranially to establish orthotopic GBM PDX models. A-D) HROG06 31d post injection of 2 × 10^5^ GBM cells, A & C) whole brain section for assessment of tumor volume and localization, B & D) 100× magnification for assessment of invasive growth of GBM cells into surrounding tissue; E & F) HROG59 34d post injection of 8.7 × 10^5^ GBM cells, F) 100x magnification; G & H) HROG59 34d post injection of 3.5 × 10^5^ GBM cells, H) 100x magnification. [file 12967_2017_1128_MOESM4_ESM.pptx]

## Slide 1
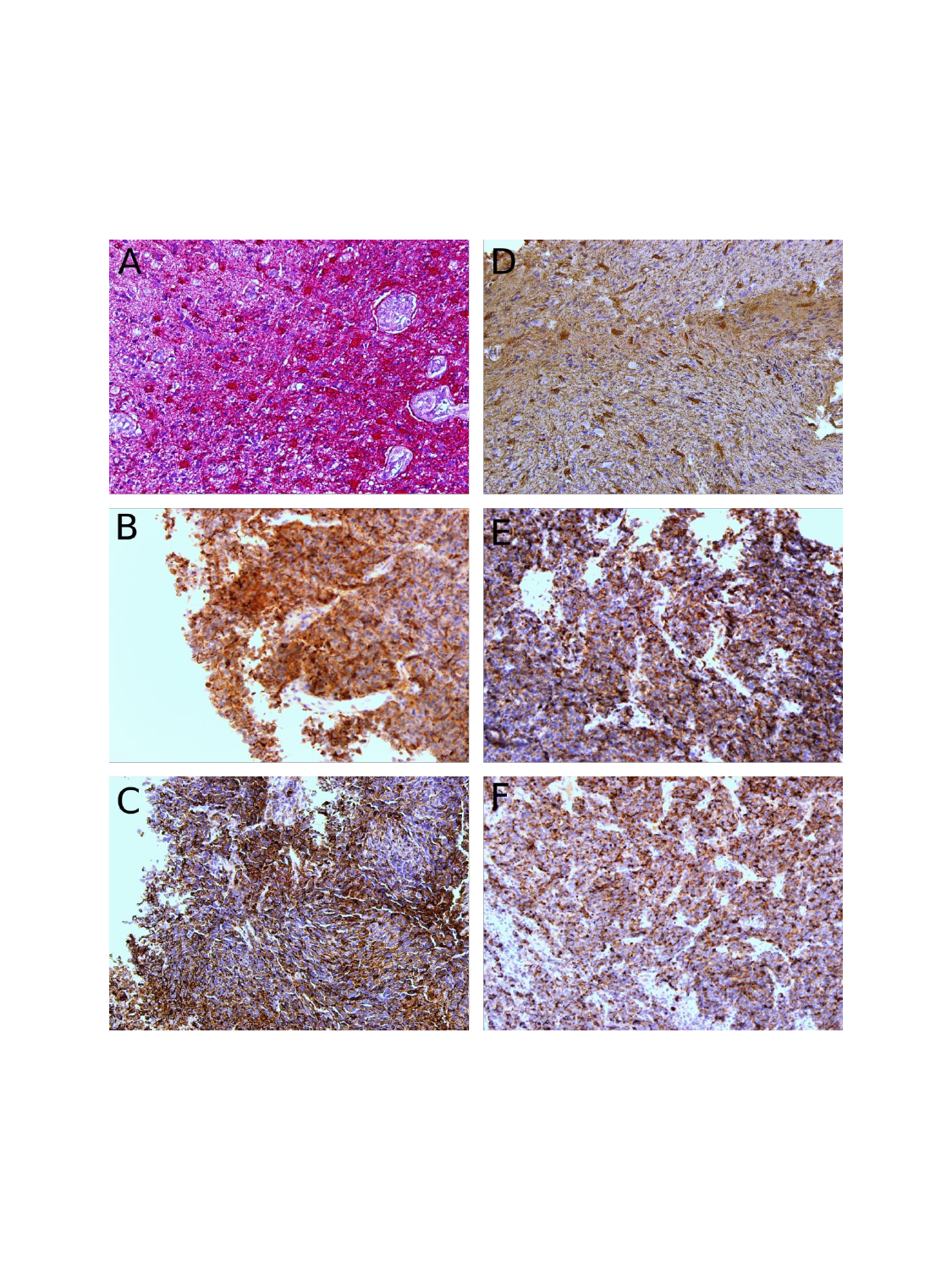

Supplement: Supplementary file 5 — Additional file 5. GFAP Immunohistochemistry staining of paraffin embedded GBM and PDX tissue sections. A) HROG33 primary GBM, stained with new fuchsine B) HROG33 PDX after first in vivo transfer, C) HROG33 PDX after second in vivo transfer, D) HROG59 primary GBM, E) HROG59 PDX after first in vivo transfer, F) HROG59 PDX after second in vivo transfer. 200-fold magnification B-F were stained with 3,3′-Diaminobenzidine. [file 12967_2017_1128_MOESM5_ESM.pptx]
